# Upcycling Fermented Adlay Bran Ethanol Extract Residues Promotes Human Dermal Fibroblast Proliferation and Wound Healing

**DOI:** 10.4014/jmb.2511.11014

**Published:** 2026-01-18

**Authors:** Ji Yun Van, Kyoung Mi Moon, Yerin Seong, Seungjin Jeong, Suhyeon Baek, Minsup Lee, Sang Gil Lee, Chun Whan Choi, Bonggi Lee

**Affiliations:** 1 Department of Smart Green Technology Engineering, Pukyong National University, Busan 48513, Republic of Korea; 2 Department of Food and Nutrition, Pukyong National University, Busan 48513, Republic of Korea; 3 Natural Product Research Team, Biocenter, Gyeonggido Business and Science Accelerator, Gyeonggi-Do, Republic of Korea; 4 Department of Molecular & Cellular Physiology, Louisiana State University Health Shreveport, Shreveport, Louisiana, USA

**Keywords:** Adlay bran, Wound healing, Fibroblast proliferation, Uridine, Deoxythymidine, Skin regeneration

## Abstract

Wound healing, critical for skin recovery after surgery, trauma, and chronic damage, proceeds through inflammation, proliferation, and remodeling phases. In this study, we focused on the proliferative phase using fermentation extracts from the residuals of Adlay bran ethanol extraction and their solvent fractions. Among them, the butanol fraction exhibited the strongest antioxidant activity and significantly enhanced the proliferation of human dermal fibroblasts, as confirmed by a real-time wound closure assay. Cell cycle analysis revealed an increased proportion of cells in the S-phase and upregulation of Cyclin A1 and Cyclin B2 mRNA, indicating accelerated progression toward the G2/M phase. We then purified the butanol fraction using C18 MPLC and HPLC and identified uridine and deoxythymidine as the main components by LC-MS/MS and NMR analysis. These nucleosides derived from fermented adlay bran residues directly promote fibroblast proliferation, highlighting their potential as sustainable bioactive compounds for skin regeneration and functional ingredient development.

## Introduction

Wound healing plays an essential role in the recovery of damaged skin from surgical procedures, traumatic injuries, and chronic diseases. This is a complex biological mechanism that progresses through inflammatory, proliferative, and remodeling stages [1, 2]. Immediately after injury, hemostasis occurs through fibrin coagulation and vasoconstriction, marking the beginning of the inflammatory phase [3]. During this phase, inflammatory cells, including neutrophils, macrophages, and lymphocytes migrate to the wound site, initiating an inflammatory response [4]. The migrated inflammatory cells, along with destroyed platelets and other cells, produce inflammatory mediators such as platelet-derived growth factor (PDGF) and transforming growth factor-β (TGF-β), stimulating fibroblasts and endothelial cells. Stimulated fibroblasts and endothelial cells migrate to the wound site, initiating the proliferative phase [4-6]. During the proliferative phase, particularly at the wound site, fibroblasts become activated and commence differentiation [7]. Fibroblasts undergo rapid mitosis and synthesize collagen, fibronectin, and proteoglycans, components of tissue remodeling, while also producing matrix metalloproteinases (MMPs) and proteolytic enzymes that aid in removing unwanted substances during wound healing [8, 9]. Moreover, TGF-β - stimulated fibroblast differentiates into myofibroblasts and produces a new extracellular matrix (ECM), essential for tissue repair [5, 6]. Myofibroblasts, called contractile cells, play a pivotal role in wound closure and promoting tissue repair during the tissue remodeling phase. This complex process repeats continuously until the damaged tissue is restored [10].

Annually, a global agricultural output exceeding five billion metric tons results in the production of agricultural residues. Notably, a substantial proportion of these residues, especially in Asian countries, are burned [11]. Several studies highlight the adverse consequences of burning crop residues, linking it to reduced air quality and health hazards [12]. This study aims to improve the environmental sustainability and economic viability of utilizing agricultural by-products.

Among these, cereal bran represents a major by-product that has been extensively investigated for its nutritional and functional properties. In particular, rice bran oil supplementation has been shown to attenuate oxidative stress and improve cardiovascular risk biomarkers in older adults with prehypertension [13], while wheat bran and its derived beverages have also been reviewed for their technological, functional, and sustainable potential [14]. Moreover, fermentation of cereal bran has been reported to provide additional health benefits, further enhancing its bioactive potential [15, 16].

Within this context, adlay bran (*Coix lacryma-jobi bran*), classified as an annual or perennial herb within the Gramineae family [17], contains various fractions, such as Adlay seed, hull, testa, and bran. Among these fractions, adlay bran (*Coix lacryma-jobi bran*) is renowned for its health benefits. It has been reported that fermenting adlay bran enhances its anti-wrinkle properties [18] and anti-melanogenic effects [19].

More importantly, while numerous studies have explored the nutritional and bioactive potential of ethanol extracts from various grains, including adlay bran, our research focuses on the residual biomass remaining after ethanol extraction, thereby further advancing the valorization of underutilized agricultural by-products. Accordingly, this study aims to enhance the post-extraction utility of these residues and to investigate the bioactive potential of fermentation extracts from the residuals of Adlay bran ethanol extraction (FRA) in promoting skin regeneration. Specifically, we will evaluate the effectiveness of fractions derived from these fermented residues in promoting skin regeneration and compare their wound healing capabilities with those of *Centella asiatica* (*C. asiatica*), a medicinal plant indigenous to South Asia. This analysis will contribute to the identification of a new resource for wound healing applications [20, 21].

## Materials and Methods

### Preparation of Fermentation Extracts from the Residuals of Adlay Bran Ethanol Extraction

The fermented adlay bran residue extract was prepared by fermenting and extracting the residue left after ethanol extraction of adlay bran. Specifically, 1 ton of adlay bran was treated with 1 ton of 70% ethanol and extracted for 24 h, followed by filtration and concentration. The residue remaining after ethanol extraction was fermented with 1% (w/w) of *Lactobacillus plantarum*, then filtered and concentrated to obtain the fermented adlay bran residue extract. This extract was subsequently suspended in water at a 1:5 ratio and partitioned with hexane at a 1:5 ratio to obtain the hexane fraction. Further partitioning was performed with ethyl acetate at a 1:5 ratio to obtain the ethyl acetate fraction. Additionally, partitioning with butanol at a 1:5 ratio yielded the butanol fraction, and the remaining aqueous layer was designated as the water fraction.

### Cell Culture

Human dermal fibroblast cells from the Hs68 cell line (American Type Culture Collection, USA) were cultured in Dulbecco’s modified Eagle’s medium supplemented with 10% heat-inactivated fetal bovine serum and 1% penicillin-streptomycin in a humidified atmosphere of 5% CO_2_ and 95% air at 37°C.

### DPPH Radical Scavenging Activity Assay

The free radical scavenging activity of the FRA and its fractions were determined using a previously published method [22]. Briefly, we investigated its capability to neutralize free radicals using 2,2-diphenyl-1-picrylhydrazyl (DPPH). 5 μl of FRA and its fractions were added to a 96-well plate containing 245 μl of 0.1 mM DPPH solution. After 30 min at room temperature, the absorbance was determined at 517 nm using a microplate spectrophotometer (AMR-100, China).

### Cell Viability Assay

Cell viability was measured using a previously established 3-(4,5-dimethylthiazol-2-yl)-5-(3-carboxymethoxyphenyl)-2-(4-sulfophenyl)-2H-tetrazolium (MTS) assay (CellTiter96 AQueous One Solution Cell Proliferation Assay Kit, Promega, USA) [23]. HS68 cells (8 × 10^3^ cells/well) were seeded in 96-well plates. After incubation, cells were incubated with a fresh medium supplemented with various concentrations of FRA (0–1,600 μg/ml) and its fractions (0–200 μg/ml) for 24 h. After 24 h, 5 μl of MTS reagent was added to each well, and the color was developed at 37°C for 1 h. The absorbance was determined at 490 nm using a microplate reader.

### Wound Healing Assay

Human fibroblast HS68 (6 × 10^3^ cells) were seeded into a Culture-Insert 2 Well in μ-Dish (IB 80206, ibidi GmbH, Germany) and placed in a 35-mm culture dish. After cell attachment for 24 h, the Culture-Insert was removed to create a cell-free gap for observation of cell migration. For *in vitro* wound healing assays, medium diluted with 200 ppm FRA was replaced and photographed under a microscope every 6 h to monitor cell migration into the interval. The rate of gap closure due to cell migration was calculated from the cell gap area through image j, with relative cell migration area (%) = (1 - GA_t_/GA_0_) × 100, in which GA_0_ is the initial gap area and GA_t_ represents the gap area at time t [24].

### Cell Cycle Analysis

Cell cycle analysis was determined using the Muse Cell Analyzer (Luminex, USA) according to the manufacturer's instructions. Briefly, Hs68 cells were seeded in 35 mm plates at a density of 1 × 10^4^ cells/well. After 24 h, cells were incubated with 200 ppm of the butanol fraction of fermentation extracts from the residuals of Adlay bran ethanol extraction (FRA-Bu) for 18 or 24 h. Cells were harvested using trypsin-EDTA and fixed in 70% ethanol at -20°C. After 3 h, cells were washed with phosphate-buffered saline (PBS) and resuspended in 200 μl of propidium iodide (PI) containing Muse Cell Cycle Reagent (MCH100106, Merck Millipore). After incubation at room temperature for 30 min in the dark, PI levels were detected to determine the percentage of cells in each cell cycle phase [25].

### Real-Time Quantitative Reverse Transcription PCR Analysis

Total cellular RNA from Hs68 cells was extracted with RiboEXTM reagent (GeneAll, Republic of Korea), and 1 μg of total RNA was used for cDNA synthesis by using a SmartGene Compact cDNA Synthesis Kit (SMART GENE, Republic of Korea). The quantitative real-time PCR was performed using the TOPreal SYBR Green qPCR PreMIX (Enzynomics, Republic of Korea) and QuantStudio 1 Real-Time PCR apparatus (Applied Biosystems, USA). The levels of expression for target genes were determined utilizing the 2^−ΔΔCT^ method [26].

### Isolation and Structural Elucidation of Uridine and Deoxythymidine from the FRA-Bu

Pre-coated Thin Layer Chromatography (TLC) plates, Silica gel 60 F*254* and Silica gel 60 RP-18 F_254_S, were purchased from Merck (Germany). For column chromatography, ODS-A (12 nm, S-7 μm, YMC GEL, Japan) were used as stationary phases. An analytical High-Performance Liquid Chromatography (HPLC) column (YMC-Pack ODS-A, 5 μm, 250 × 4.6 mm I.D.) was obtained from YMC.

Extra Pure (EP) grade solvents used for extraction, fractionation, and column chromatography were sourced from Samchun and Daejung Chemical Co., Ltd. (Republic of Korea). HPLC grade solvents for analysis were purchased from Burdick & Jackson (USA). Deuterated solvents for Nuclear Magnetic Resonance (NMR) analysis were obtained from Cambridge Isotope Laboratories, Inc. (USA). The Pre-coated TLC visualization reagent was a solution of vanillin in 10% sulfuric acid in ethanol.

^1^H and ^13^C-NMR spectra were recorded on a Bruker Advance spectrometer (700 MHz, Germany). Preparative HPLC was performed on an LC-8A system (Shimadzu, Japan), and analytical HPLC was conducted using an Agilent 1200 series system (USA). Medium Pressure Liquid Chromatography (MPLC) was carried out on a CombiFlash Rf system (Teledyne Isco, USA). A UV scanning system (Uvitec Cambridge, USA) was used for visualization at 254 nm and 365 nm. Solvents were removed using a rotary vacuum evaporator (Rotavapor N-1000, NVC-2100, Eyela, Japan) in conjunction with a cold trap bath (CA-1112, Eyela). Final samples were dried using a freeze dryer (FD8508, Ilshin Lab Co., Ltd., Republic of Korea).

### Statistical Analysis

Statistical comparisons were conducted using GraphPad Prism 5.0 (GraphPad Software, USA). All experiments were performed in triplicate or more, and results are presented as mean ± SEM. Comparisons between two groups were analyzed by unpaired t-test, while multiple group comparisons were analyzed by one-way ANOVA followed by Tukey’s or Dunnett’s post hoc test, as indicated in the figure legends.

## Results

### Extraction Efficiency of Different Fractions

The extraction yield of various fractions extracted from FRA was conducted to determine the most effective method for obtaining high yields from adlay bran residue. The results indicated that the butanol fraction exhibited the highest yield. Specifically, the butanol fraction accounted for 10% of the total residue, which is substantially higher compared to the yields of the Hexane fraction (0.5%), Ethyl acetate fraction (2.3%), and Water fraction (4%). These notable yields emphasize the superior efficiency of butanol in extracting bioactive compounds relative to the other solvents used in the study. This efficiency is likely due to the higher polarity of butanol, which allows it to dissolve a wider range of polar bioactive compounds present in the adlay bran residue, thereby resulting in a higher extraction yield (Fig. S1).

### Antioxidant Activity of FRA and Its Fractions

**Fig. 1:**
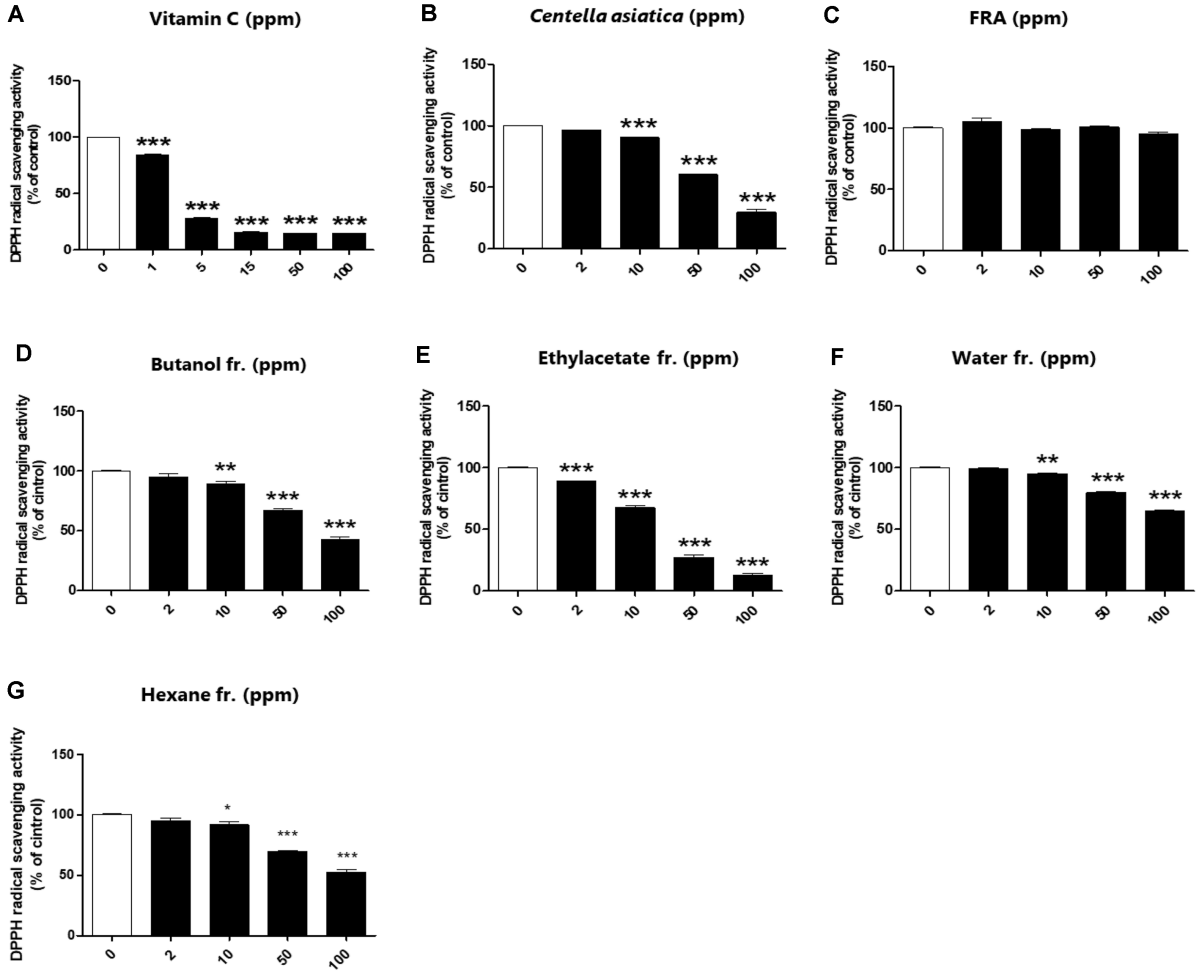
Antioxidant effect of FRA and its fractions through inhibition of free radical production. The DPPH inhibitory activity of FRA and its fractions (0–100 μg/ml) was tested using a spectrophotometer at 510 nm. Vitamin C was used as a positive control. Data are presented as mean ± standard error (*n* = 3). Significant differences were analyzed using one-way ANOVA and Dunnett's test. *p < 0.05, **p < 0.01, ***p < 0.001 compared with the untreated group.

In wound healing, reactive oxygen species are recognized to cause transient inflammatory processes and to delay tissue repair [27]. Therefore, we confirmed the free radical scavenging activity of FRA and its fractions using 2,2-diphenyl-2-picrylhydrazyl. Vitamin C, a potent antioxidant, served as the positive control in this study. After mixing the sample and DPPH solution and standing at room temperature in the dark for 30 min, the mixed sample was measured at 517 nm absorbance. Vitamin C showed a strong antioxidant effect with an inhibition rate of 80% (Fig. 1A). FRA showed no significant antioxidant effect (Fig. 1C), but the antioxidant potency of the fractions varied depending on the fractionation method. In particular, both butanol and ethyl acetate fractions showed antioxidant potential exceeding 50%, which is similar to the well-known antioxidant properties of *C. asiatica* (Fig. 1B-1G) [28, 29].

### Effects of FRA and Its Fractions on Fibroblast Proliferation without Cytotoxicity

**Fig. 2:**
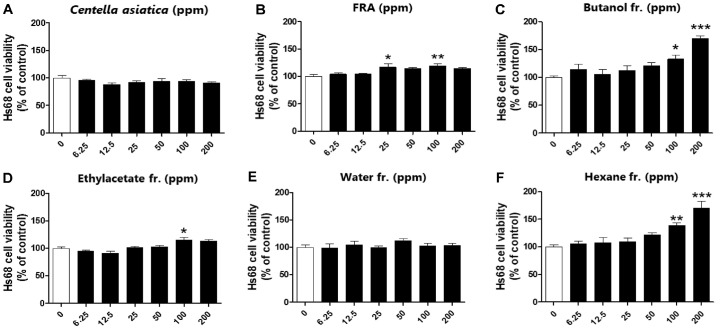
FRA and its fractions increase fibroblast proliferation without cytotoxicity. Hs68 cells were treated with different concentrations of FRA (0– 1,600 μg/ml) and its fractions (0–200 μg/ml) for 24 h and then cytotoxicity was performed (*n* = 6). Data are presented as mean ± standard error (*n* = 3). Significant differences were analyzed using one-way ANOVA and Dunnett's test. *p < 0.05, **p < 0.01, ***p < 0.001 compared to the untreated group.

Fibroblasts play an important role in repair from the late inflammatory phase of the wound healing process through to the final epithelialization of damaged tissue [4-6]. In this study, the effects of FRA and its fractions on cell proliferation were investigated using the Hs68 human fibroblast cell line. MTS assay was performed to confirm the cell proliferation effect of FRA and its fractions in Hs68 cells. As a result, *C. asiatica*, which is effective in wound healing, had no proliferative effect, while cells treated with FRA showed a proliferative effect in a concentration-dependent manner (Fig. 2A and 2B). In particular, butanol extract significantly increased cell proliferation by more than 50% at a concentration of 200 ppm among various extracts (Fig. 2C-2F).

### FRA-Bu 200 ppm Increases the Migration and Proliferation of Hs68 Fibroblasts

**Fig. 3:**
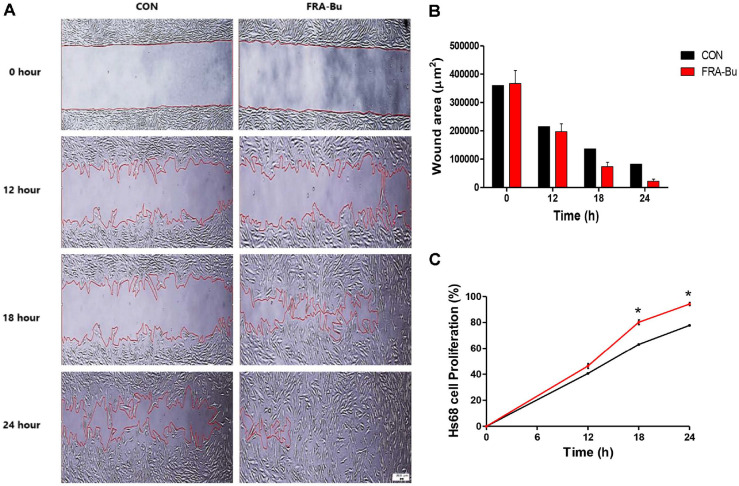
Effects of FRA-Bu 200 ppm on proliferation and adhesion of Hs68 fibroblasts. (**A**) The area and speed of Proliferation of Hs68 cells was measured through a 500 μm cell-free gap created by a culture insert placed in the center of a 35 mm μ-Dish (Ibidi, Germany). Measurements were made using a microscope every 6 h during 24-h incubation through real-time monitoring. Scale bar = 200 μm. (**B**) The wound area of Hs68 cells treated with 200 ppm of FRA-Bu was significantly narrowed compared to untreated cells. (**C**) The migration area expressed as a percentage based on the initial cell-free interval, increased significantly at 18 h of culture. The area area was measured in real-time using image j. Significant differences were analyzed using by unpaired *t*-test (*n* = 3). Data are presented as mean ± standard error (*n* = 3). *p < 0.05 compared with the untreated group.

The efficacy of the FRA-Bu was visualized using 24-well plates with inserts, demonstrating its capacity to stimulate fibroblast proliferation and migration. As shown in Fig. 2, the presence of 200 ppm FRA-Bu demonstrated fibroblast movement within consistent non-cellular intervals, indicating the anticipated effects of FRA on wound healing. Cells cultured with FRA-Bu 200 ppm exhibited complete potential wound closure in 24 h compared to untreated cells (Fig. 3A). Particularly, cells treated with FRA-Bu at the artificial wound site demonstrated maximum stimulation effect 18 h post-treatment (Fig. 3B). The effect of the extract on cell migration and proliferation was expressed as a percentage of wound closure (Fig. 3C).

### Cell Migration is Affected by Cell Cycle Regulation of FRA-Bu

**Fig. 4:**
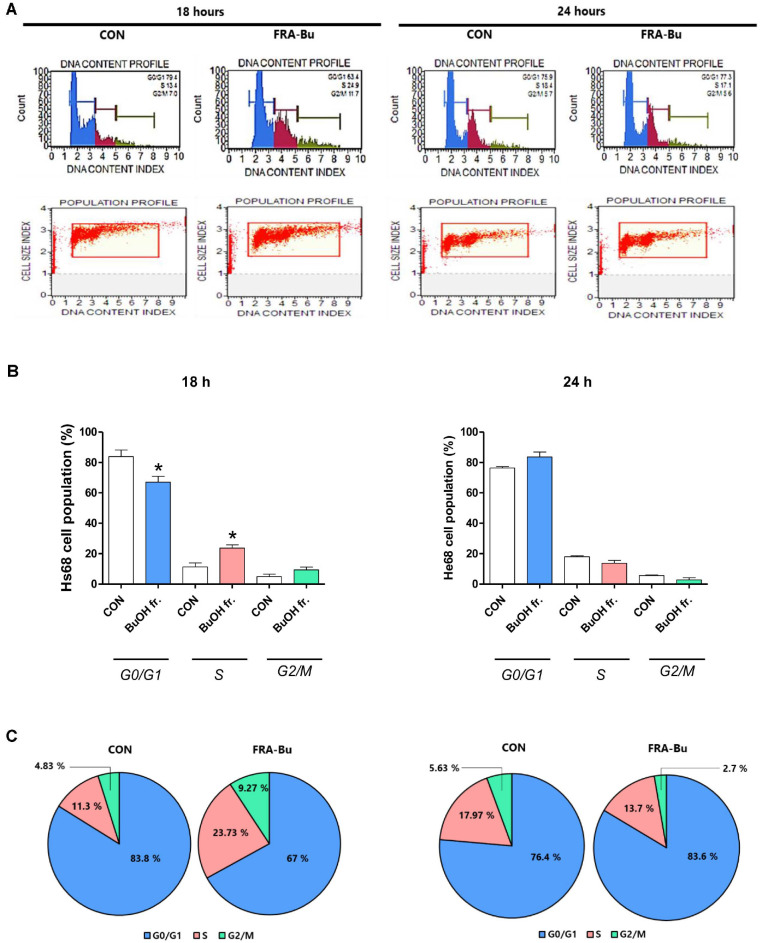
Cell proliferation is influenced by the cell cycle. The cell ratio in the G0/G1, S, and G2/M phases of proliferation on the FRA-Bu treated Hs68 cells was analyzed using Muse Cell Cycle Analyser. (**A**) A histogram of a representative experiment shows the effect of FRA-Bu on the cell cycle profile. (**B-C**) In particular, the treated cells showed a significant increase in the S phase of the cell cycle, the origin of DNA replication, after 18 hours of culture. Significant differences were analyzed using an unpaired *t*-test (*n* = 3). Data are presented as mean ± standard error (*n* = 3). *p < 0.05 compared to the untreated group.

To evaluate the impact of FRA-Bu on the cell cycle dynamics of fibroblast proliferation, we performed an analysis of cell distribution across the various phases (G0/G1, S, and G2/M) using flow cytometry. We utilized the Muse cell cycle kit to obtain images and quantification of cell cycle dynamics (Fig. 4A). Treatment with 200 ppm of FRA-Bu resulted in a significant modulation of the cell cycle: the proportion of cells in the S phase doubled from 11.3% to 23.7%. This increase was accompanied by a reduction in the G0/G1 phase population from 83.8% to 67% and an increase in the G2/M phase population from 4.8% to 9.3% (Fig. 4B and 4C). These results indicate a potential role for FRA-Bu in enhancing fibroblast proliferation through the induction of S phase entry.

### Increased mRNA Levels of Cyclin A2 and Cyclin B2 in FRA-Bu-Treated Hs68 Cells

**Fig. 5:**
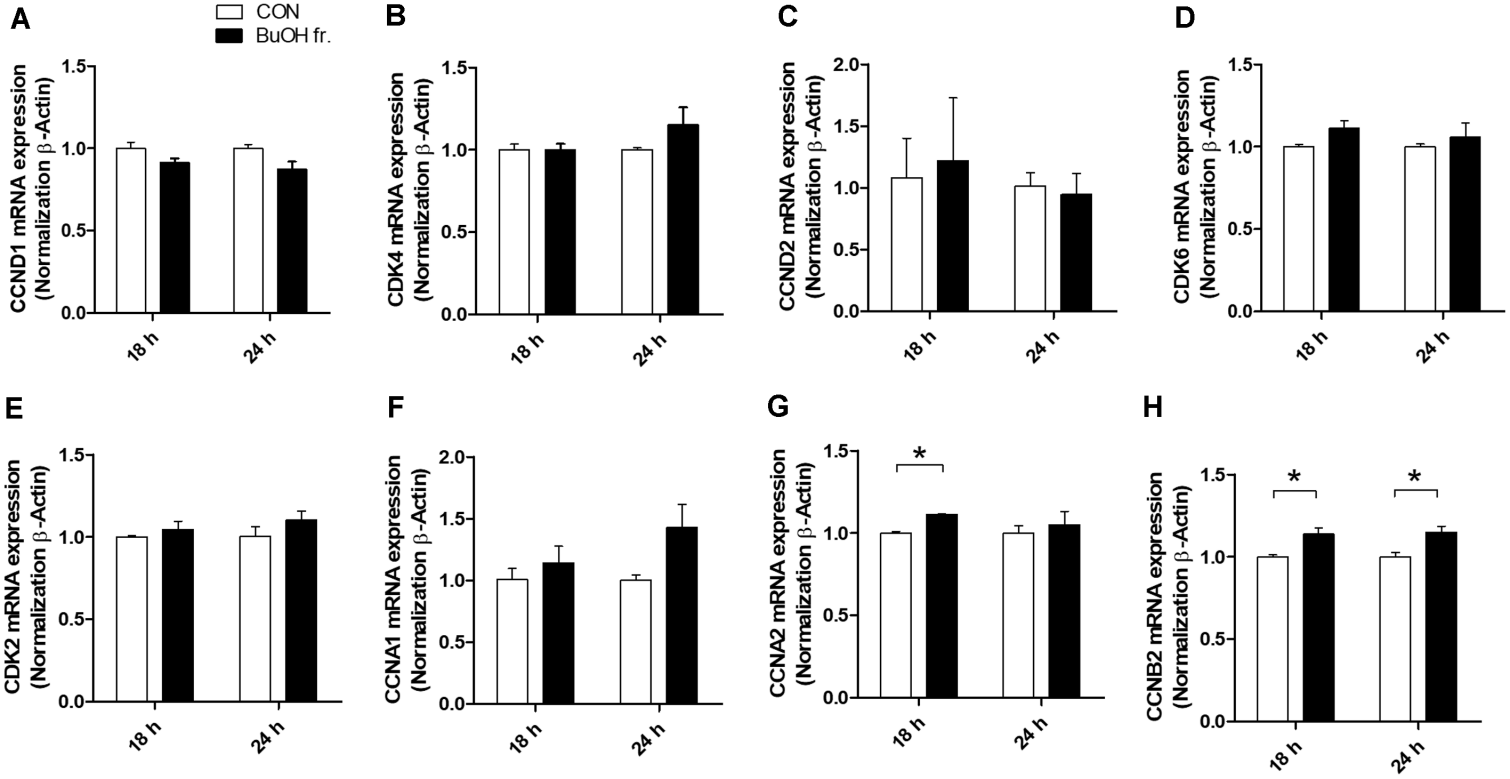
Increased mRNA levels of Cyclin A2 and Cyclin B2 in FRA-Bu-treated Hs68 cells. The mRNA expressions of *CCND1*, *CDK4, CCND2, CDK2, CCNA1*, *CCNA2*, and *CCNB2* were measured by qPCR in Hs68 cells (treated or nontreated) after 18 and 24 h. Significant differences were analyzed using an unpaired *t*-test (*n* = 3). Data are presented as mean ± standard error (*n* = 3). *p < 0.05 compared to the untreated group.

Previous studies aimed at identifying cell cycle regulation mechanisms have established that Cyclin D1 (*CCND1*), Cyclin-dependent kinase 4 (*CDK4*), Cyclin D2 (*CCND2*), Cyclin-dependent kinase 2 (*CDK2*), Cyclin A1 (*CCNA1*), Cyclin A2 (*CCNA2*), Cyclin B2 (*CCNB2*), and other molecules are recognized as indicators of cell proliferation[30]. In our study, flow cytometry showed that induction of the S phase was associated with increased mRNA expression levels of *CCNA2* and *CCNB2* (Fig. 5). *CCNA2* and *CCNB2* function as central regulators of the cell cycle, specifically promoting cell proliferation and progression through the S and G2 phases [31, 32]. *CCNA2* is essential for DNA replication and entry into mitosis, while *CCNB2* plays a crucial role not only in the mitotic phase but also in regulating the transition from G2 to M phase by activating *CDK1* [31, 32]. These findings imply a potential role for FRA-Bu as an innovative therapeutic agent in wound healing, as it augments DNA synthesis in human fibroblasts, thereby promoting swift cell migration and proliferation. Moreover, the heightened expression levels of *CCNA2* and *CCNB2* mRNA observed in our study underscore their significance in driving cell cycle advancement, particularly during the S and G2 phases, suggesting their potential utility as targets for modulating cellular proliferation in diverse therapeutic contexts.

### Isolation and Structural Elucidation of Uridine and Deoxythymidine from the FRA-Bu

The isolation of nucleoside compounds from the butanol fraction of fermented adlay bran was achieved using C18 MPLC. As illustrated in Fig. S1, the butanol fraction was fractionated into five primary fractions (F1–F5). Fractions F1 and F5 were further purified by HPLC and eluted with H_2_O in methanol in a step-gradient manner, from 1% to 50%. F14 and F55 was also purified by a similar manner with RP-18 column chromatography eluted with methanol in H_2_O (1% to 50%) in a stepwise gradient (Table S1).

**Fig. 6:**
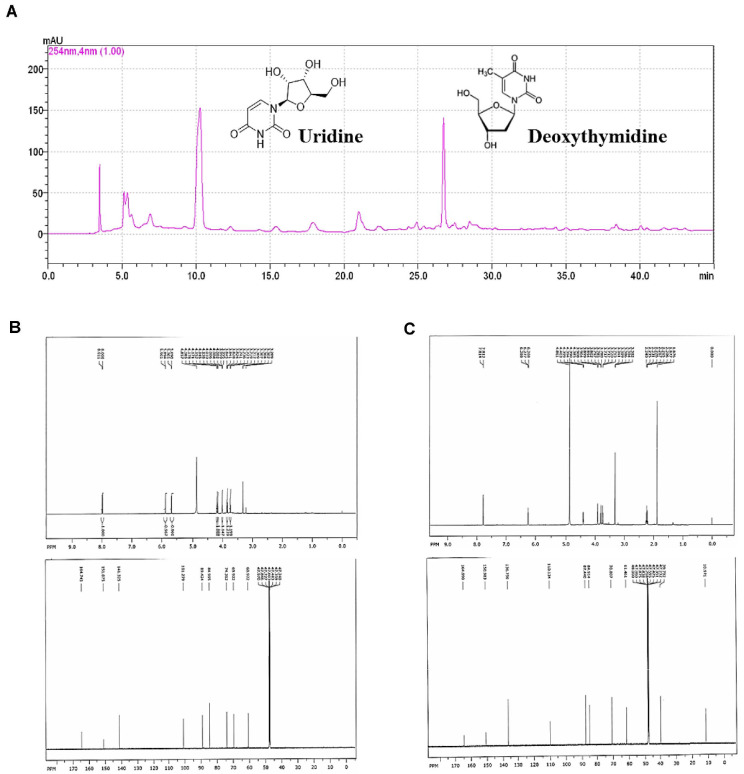
HPLC and NMR-based identification of uridine and deoxythymidine in FRA-Bu. (**A**) HPLC chromatogram of FRA-Bu on a C18 column at 254 nm, with major peaks at ~9.5 and ~26.5 min assigned to uridine and deoxythymidine (structures above peaks). (**B–C**) ^1^H and ^13^C NMR spectra of the compounds isolated from fractions F1 and F5, confirming their structures as uridine and deoxythymidine, respectively.

These fractions were subsequently analyzed by NMR spectroscopy to determine their major components. In parallel, chemical profiling of the butanol extract was conducted using HPLC monitored at four UV wavelengths (210, 254, 280, and 330 nm). The resulting chromatograms (Fig. 6A) displayed distinct peaks at approximately 9.5 and 26.5 min, consistently detected at 254 nm, which is indicative of UV-absorbing nucleoside compounds. Structural confirmation was achieved by ^1^H and ^13^C NMR spectroscopy (Fig. 6B and 6C): the spectra of the compound from fraction 1 aligned well with uridine, supporting its identification as a ribose-linked pyrimidine nucleoside, while fraction 5 exhibited signals characteristic of deoxythymidine, confirming its identity as a deoxyribonucleoside. Collectively, these results demonstrate that uridine and deoxythymidine were successfully isolated and structurally identified from the butanol fraction of fermented adlay bran using HPLC and NMR-based analytical techniques.

## Discussion

Globally, agricultural activities produce a substantial amount of residue annually, with a significant portion incinerated, particularly in Asia [11]. This practice contributes to environmental issues, including poor air and water quality, which pose health risks [12]. Therefore, our study explores the potential of utilizing these residues to not only address environmental sustainability but also to extract new bioactive compounds. This dual approach underscores the valorization of agricultural by-products, positioning fermented adlay bran residues as a promising source of bioactive nucleosides for sustainable food, cosmetic, and nutraceutical applications.

In this study, the extraction yields of various FRA fractions were investigated and compared. The results showed that the butanol fraction accounted for 10% of the total residue, demonstrating the highest yield. These findings suggest that butanol is more effective than other solvents in extracting bioactive compounds from adlay bran residue. We highlight the therapeutic potential of FRA-Bu in modulating the critical fibroblast proliferative phase of wound healing. Fibroblasts play a crucial role in wound recovery by rapidly proliferating to synthesize extracellular matrix proteins and glycoproteins and promoting angiogenesis essential for tissue repair [33]. Our results demonstrate that FRA-Bu significantly enhances both the proliferation of fibroblasts (Figs. 2 and 3). Specifically, FRA-Bu increased the fibroblast population in the S phase of the cell cycle, where DNA replication is primarily activated, thereby boosting cellular proliferation rates (Figs. 4 and 5). This underscores the pivotal role of FRA-Bu in facilitating fibroblast progression through the cell cycle, enhancing tissue restoration and regeneration processes.

Metabolic changes, such as increased levels of pyrimidine nucleosides including uridine, have been reported in highly regenerative tissues and human stem cell models, and are associated with enhanced cell proliferation and tissue repair [34-36]. Deoxythymidine is also a key pyrimidine precursor directly used for DNA synthesis. Disruption of the dNTP pool, including dTTP, has been reported to delay S-phase progression and induce replication stress [37, 38]. Overall, the uridine and deoxythymidine detected in FRA-Bu may have contributed to a metabolic environment that supports cell-cycle progression and cell proliferation.

Moreover, our findings provide mechanistic insights into the molecular basis of the proliferative effects induced by FRA-Bu. Elevated mRNA expression levels of *CCNA2* and *CCNB2*, key regulators of the cell cycle, were observed, indicating that FRA-Bu actively promotes the transition through the S and G2 phases of the cell cycle (Fig. 5G and 5H). This promotion of cell cycle progression is crucial for efficient DNA synthesis and mitotic entry, further emphasizing FRA-Bu's potential as an effective therapeutic agent in wound healing strategies. The enhanced expression of these cyclins supports their role in driving the rapid proliferation and timely migration of fibroblasts, essential for effective wound closure and healing.

Furthermore, our study evaluated the efficacy of FRA-Bu compared to *C. asiatica*, a well-known medicinal plant used in wound healing. Numerous studies have shown that *C. asiatica* primarily enhances wound healing through its anti-inflammatory properties and by promoting the synthesis of collagen and other extracellular matrix components, rather than directly stimulating fibroblast proliferation [39-42]. In contrast, our findings show that FRA-Bu directly stimulates the cell cycle and promotes fibroblast proliferation. This suggests that FRA-Bu offers a complementary therapeutic approach that includes both anti-inflammatory and proliferative effects.

Thus, FRA-Bu demonstrates potential not only as a therapeutic agent for enhancing wound healing but also as a practical example of valorizing agricultural by-products in medical science. Further studies are essential to fully elucidate the safety criteria of FRA-Bu and its long-term effects in clinical applications.

## Conclusion

This study demonstrates that the FRA-Bu promotes skin cell proliferation and cell cycle progression in human dermal fibroblasts. FRA-Bu showed the highest extraction yield (~10%) among the solvent fractions tested, and chromatographic purification revealed uridine and deoxythymidine as major nucleoside constituents. These compounds are known to enhance DNA repair and support tissue regeneration, suggesting that FRA-Bu exerts dual functional effects.

Taken together, these findings indicate that FRA-Bu is a promising natural bioactive fraction for supporting skin regeneration. Moreover, this study highlights the potential of upcycling agricultural by-products into value-added functional ingredients. Further work on safety, stability, and formulation could facilitate the incorporation of FRA-Bu into cosmetic or functional food applications.

## Supplemental Materials

Supplementary data for this paper are available on-line only at http://jmb.or.kr.
