# Supplementary material for: Upcycling Fermented Adlay Bran Ethanol Extract Residues Promotes Human Dermal Fibroblast Proliferation and Wound Healing
Source: J Microbiol Biotechnol. 2026 Jan 18;36:e2511014. doi: 10.4014/jmb.2511.11014 (PMC12828326; doi:10.4014/jmb.2511.11014)
Supplement: Supplementary file 1 [file jmb-36-e2511014-supple.pdf]

J. Microbiol. Biotechnol. 2026. 36: e2511014
https://doi.org/10.4014/jmb.2511.11014

Supplementary Figure and Table

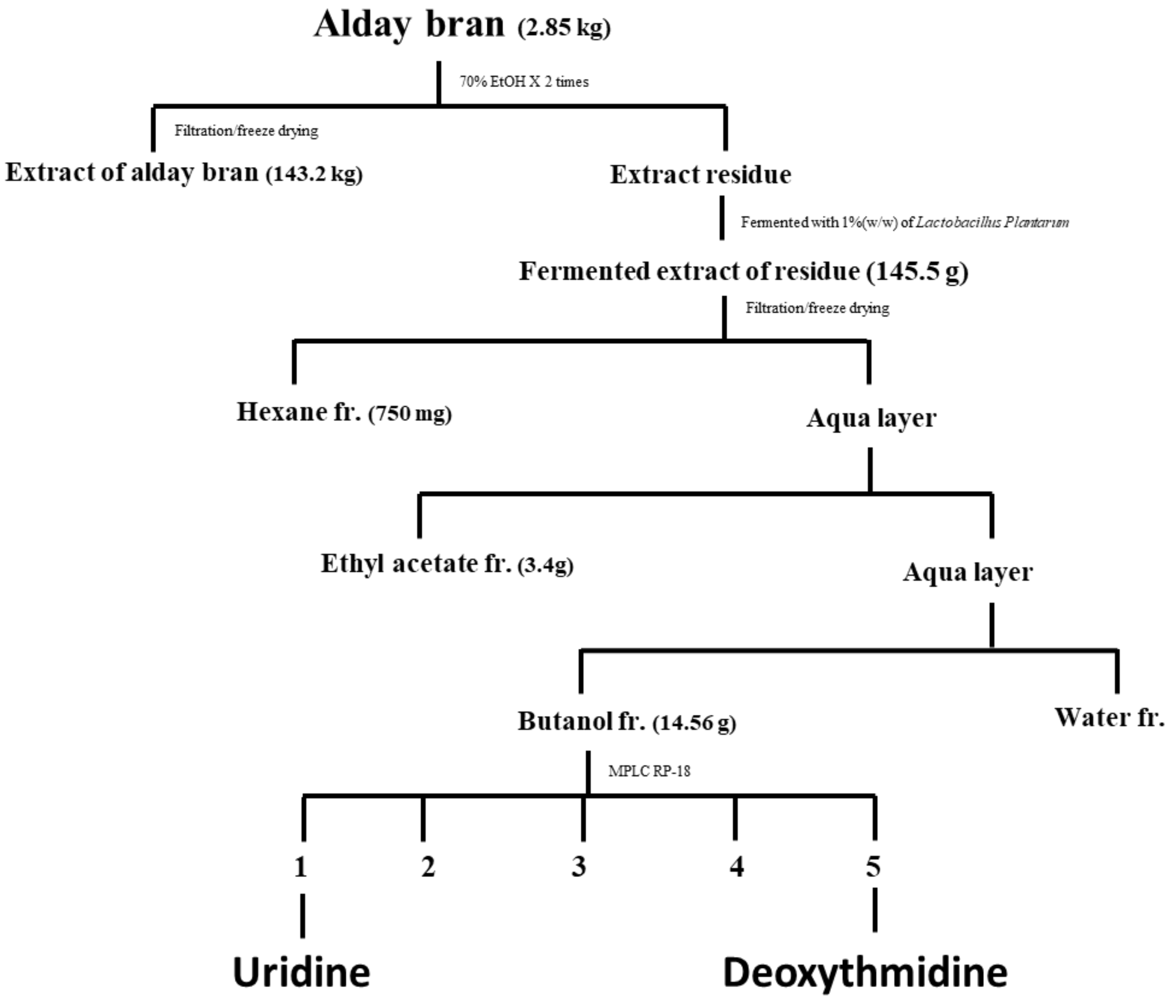

Fig. S1. Preparation of FRA and its fractions. Adlay bran residues were extracted with ethanol, fermented, and then fractionated into hexane, ethyl acetate, n-butanol, and water fractions. The butanol fraction (FRA-Bu) was further separated on an RP-18 MPLC column into five fractions (1–5), from which uridine (fraction 1) and deoxythymidine (fraction 5) were isolated. Extraction yields and detailed chromatographic conditions for each fraction are summarized in Supplementary Table 1, and representative chromatograms of the nucleoside-containing fractions are shown in Fig. 6.

Table. S1

1. HPLC analysis conditions

1-1. Reagent & Apparatus

Reagent & Apparatus	Manufacture, Grade
Methanol	B&J Honeywell / HPLC grade
0.05% TFA water	Daejung / HPLC grade

1-2. Instrument

Instrument	Specification
HPLC systems	SHIMADZU LC-20A
- Detector	UV

1-3. Method of Analysis (HPLC)

Module	Condition		
Instrument	HPLC		
Mobile Phase	A : 0.05% TFA Water<br>B : Methanol<br>- Gradient elution		
		A	B
	init.	100	0
	0	100	0
	30	40	60
	30.01	0	100
	40	0	100
	40.01	100	0
	50	100	0
Column	YMC-Pack ODS-A (250 x 4.6 ㎜ I.D)		
Detector(Wavelength)	UV 210, 254, 280, 330 nm		
Flow rate	1.0 ㎖/min		
Injection Volumn	20 ㎕		
Column Oven Temperature	40℃		
Run Time	50 min		
